# Antitumor Effects of HPV DNA Vaccine Adjuvanted with Beclin-1 as an Autophagy Inducer in a Mice Model

**DOI:** 10.29252/ibj.23.6.388

**Published:** 2019-11

**Authors:** Hamed Naziri, Alireza Tahamtan, Maryam Dadmanesh, Mohammad Barati, Khodayar Ghorban

**Affiliations:** 1 Department of Immunology, School of Medicine, Aja University of Medical Sciences, Tehran, Iran;; 2 Department of Virology, School of Public Health, Tehran University of Medical Sciences, Tehran, Iran;; 3 Infectious Diseases Research Centre, Golestan University of Medical Sciences, Gorgan, Iran;; 4 Department of Microbiology, Golestan University of Medical Sciences, Gorgan, Iran;; 5 Department of Infectious Diseases, School of Medicine, Aja University of Medical Sciences, Tehran, Iran;; 6 Infectious Diseases Research Center, Aja University of Medical Sciences, Tehran, Iran

**Keywords:** Adjuvants, Autophagy, Beclin-1

## Abstract

**Background:**

There is a growing interest in development of an effective adjuvant system for improving DNA vaccines. Recent findings have confirmed an important role for autophagy in both innate and adaptive immunity. The current study was undertaken to determine the efficacy of autophagy induction with Beclin-1, as a novel adjuvant system, in mice immunized with human papilloma virus (HPV) DNA vaccine.

**Methods:**

To determine whether autophagy induction with Beclin-1 enhances the efficacy of HPV DNA vaccine, female C57BL/6 mice were challenged with TC-1 tumor cells and were immunized three times at one-week intervals. Two weeks after the final immunization, the mice were sacrificed, and the antitumor effects were assessed by measurement of lymphocyte proliferation, cytotoxicity, cytokine production, and tumor regression.

**Results:**

Beclin-1 in combination with HPV-16 DNA vaccine encoding the E7 antigen induced a higher level of lymphocyte proliferation and cytotoxicity than the DNA vaccine alone. The novel combination increased the production of IFN-γ and highly inhibited tumor progression in comparison with DNA vaccine alone.

**Conclusion:**

Administration of Beclin-1, as an autophagy inducer, with HPV DNA vaccine produces antitumor effects, providing an effective adjuvant for the induction of a strong antitumor immune response.

## INTRODUCTION

Human papilloma virus (HPV), a small non-enveloped double-stranded DNA virus, is known as an ethological agent of cervical cancer^[^^1^^]^. This type of cancer is one of the most important malignancy and a prominent cause of death among women in the world in underdeveloped nations^[^^2^^]^. There is evidence that HPV is involved in anal, head, and neck cancer in humans^[^^3^^]^. Although over 200 HPV genotypes have been found, only high-risk types of HPV (16, 18, 31, and 45) are associated with ~80% of cervical cancers, facilitating effective vaccine development^[^^4^^]^. Two recombinant HPV preventive vaccines with no therapeutic benefits, Gardasil and Cervarix, which carry artificially manufactured virus-like particles of the L1 epitope, have been approved by the US Food and Drug Administration^[^^5^^]^. Designing and constructing new therapeutic vaccines against HPV are important for the treatment of cervical cancer and HPV infections^[^^6^^]^.

There are several approaches to construct therapeutic HPV vaccines, such as live-vector-based, peptide/ protein, and cell-based strategies, as well as DNA vaccine^[^^2^^]^. DNA vaccines have been developed as a promising procedure for immunotherapy of cancer because it represents a valid way to generate antigen-specific immunotherapy^[^^7^^]^. DNA vaccines are composed of bacterial plasmids that can be simply prepared in a large scale with great purity. They are highly stable and offer simple effective means of inducing broad-based immunity^[^^8^^]^. To combat HPV, DNA vaccines targeting E7 oncoprotein has been considered as a potentially efficacious method due to their essential role in viral life cycle and oncogenic transformation alike^[^^9^^]^. There are some disadvantages to clinical administration of DNA vaccines. The approach suffers from low immunogenicity and restricted specificity for antigen-presenting cells^[^^10^^,^^11^^]^. In addition, the effectiveness of DNA vaccines expressing oncoproteins can be boosted by using an effective adjuvant^[^^12^^]^.

Using adjuvants to induce authophagy is a promising strategy for enhancing immune responses^[^^13^^,^^14^^]^. Autophagy is an extremely preserved and continuous biological proccess that can lead to the surrounding of cytoplasmic contents in a cup-shaped double-membrane or multi-membrane-bound structures known as an autophagosome^[^^15^^]^. Autophagy has been implicated in more efficient MHC-mediated antigen presentation of intracellular microorganisms such as viruses and bacteria^[^^16^^]^. Processing endogenous and exogenous antigens for presentation by MHC-II molecules is facilitated by autophagy for degradation by lysosomal hydrolases. Moreover, extracellular pathogens can be presented by MHC-I during cross-presentation^[^^17^^]^. The essential role of autophagy in both innate and adaptive immunity has been confirmed. Recently, it has been determined that autophagy plays a significant role in cellular immunity against intracellular pathogens such as viruses or intracellular bacteria by enclosing and targeting them for removal function through the delivery of antigens for major histocompatibility complex classes I and II (MHC-I and-II) presentation. Autophagy also takes a part in innate immune responses by the removal of pathogens and the induction of acquired immunity^[^^18^^]^. 

Studies have reported that triggering autophagy by Beclin-1 enhances the antigen-presenting cell function and augments the potency of the immune response^[^^19^^,^^20^^]^. Beclin-1 is a key factor for the activation of autophagy and plays an essential role in autophagosome formation and autophagosome/ endosome maturation^[^^21^^]^. The current study developed a novel adjuvant system to enhance the therapeutic activity of HPV-16 DNA vaccine in a mice model based on the ability of autophagy to induce immune responses. Here, we demonstrated that the administration of Beclin-1, as an autophagy inducer, with HPV DNA vaccine induces antitumor effects, providing an effective adjuvant for the induction of a strong antitumor immune response.

## MATERIALS AND METHODS

**Plasmid construction**

The construction, amplification, and purification of plasmid pcDNA3.1 expressing HPV-16 E7 have been described previously, and the expression of E7 from pcDNA3.1 was performed in CHO cells^[^^22^^,^^23^^]^. The construction, amplification, and purification of plasmid pVITRO2 expressing Beclin-1 have formerly been described, and expression of Beclin-1 from pVITRO2 was performed in HEK293 cells^[^^24^^]^. Briefly, the plasmids were generated by retrieving HPV-16 E7 and Beclin-1 sequences from the GenBank database and cloned into plasmids. Plasmid constructs were confirmed by DNA sequencing and expression. *E. coli *bacterial strain DH5α was used for propagation and preparation of plasmids. The purity and identity of the plasmid was confirmed by agarose gel electrophoresis. To evaluate the expression of genes from constructed plasmids, the proteins were separated by SDS polyacrylamide gel electrophoresis, blotted on a membrane and incubated with the specific monoclonal antibody. Details of each method and results are available elsewhere^[^^22^^-^^24^^]^.

**Immunization of mice and experimental assays**

Six-to-eight-week-old female C57BL/6 mice (Pasteur Institute of Iran, Tehran) were divided into five groups (10 mice/group), including PBS, pCDNA, pVITRO2-Beclin1, pCDNA-E7, and pCDNA-E7 + pVITRO2-Beclin1. The groups were challenged with subcutaneous injection of 2 × 10^5^ TC-1 tumor cell lines in 100 μl PBS in the right flank. After one week, the mice were immunized subcutaneously with 90 μg of vaccine on three occasions separated by 7-day intervals. The control group received a similar volume of PBS. In the combination group (pCDNA-E7 + pVITRO2-Beclin1), the mice received 90 μg of each vaccine and were sacrificed two weeks after the final immunization for experimental assay. All animal studies were approved by the Ethical Committee of AJA University of Medical Sciences (Tehran, Iran; ethical code no. 241). 

The lymphocyte proliferation assay was performed using a cell proliferation ELISA BrdU kit (Roche, Germany) through the incubation of splenocytes with the purified antigen according to manufacturer's protocol. Briefly, spleen cells (10^6^ cells/well) were cultured in RPMI and stimulated in the presence of antigen in 96-well flat-bottom plates and incubated in 5% CO_2_ at 37 °C for 72 h. Then 20 µL BrdU labeling solution was added per well and incubated in a CO_2_ incubator at 37 °C for 12 h. BrdU was detected using Brdu antibodies (Roche, Germany), and analysis was performed according to manufacturer's instructions.

The cytolytic T lymphocyte (CTL) activity was determined based on the measurement of lactate dehydrogenase release from lysed target cells using a cytotoxicity detection kit (Takara, Japan) according to the instructions provided by manufacturer. Briefly, the lymphocytes (as effector cells) were cultured in phenol red-free RPMI containing 3% fetal calf serum with EL-4 cells previously stimulated with antigen as target cells (50:1 effector-to-target cell ratio). After 4 h of incubation, the culture plates were centrifuged and the supernatants (50 µl/well) were transferred to the 96-well flat-bottom plates. Plates were read at 490 nm after 30 min incubation at room temperature, and cytotoxicity was determined as:

Cytotoxicity (%) = (Experimental value - low control/high control - low control) × 100

In the high control, all EL-4 cells were lysed with Triton X-100 and in the low control, the cells were treated only with assay medium. The production of IFN-γ and IL-4 from splenocyte culture supernatant was evaluated by ELISA using commercial IFN-γ and IL-4 mouse kits (PeproTech, USA) according to manufacturer's protocol. Briefly, an aliquot of 5 × 10^5^ cells/100 μl was added to the wells of 96-well plates. After three days of incubation with antigen, the cell supernatants were collected and used to detect the levels of IFNγ and IL-4.

**Statistical analysis**

Statistical calculations and graph preparation were performed using GraphPad Prism version 6.0 for Windows (GraphPad, USA). Lymphocyte proliferation, CTL, and cytokine assays were analyzed by one-way ANOVA. Differences were considered statistically significant at *p* < 0.05.

## RESULTS

**Lymphocyte proliferation response**

**Fig. 1:**
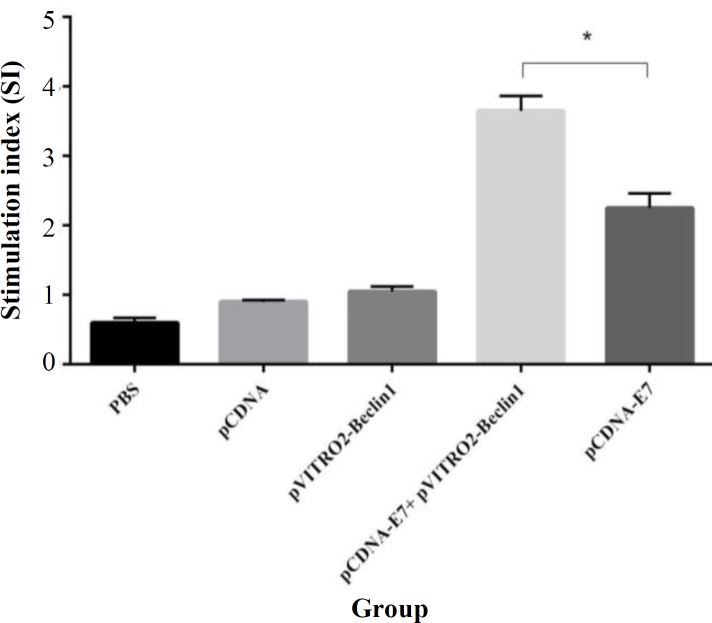
Lymphocyte proliferation response. Each group of mice was immunized three times according to different groups. Two weeks after the last immunization, mice were sacrificed, and splenocytes were obtained. Then lymphocyte proliferation was performed with cell proliferation ELISA Brdu kit. Results represented as the mean ±SD of five animals for each group.  ^*^p < 0.05

In order to perform the lymphocyte proliferation assay, splenocytes from the immunized mice were removed and restimulated *in vitro* with antigens two weeks after the final immunization. As represented in Figure 1, HPV-16 E7 DNA vaccine enhanced the proliferative response to E7 antigen when compared with the control groups (PBS, pCDNA, and pVITRO2-Beclin1). However, lymphocyte proliferation was dramatically higher in mice inoculated with HPV-16 E7 DNA vaccine adjuvanted with Beclin-1 (pCDNA-E7 + pVITRO2-Beclin1), compared to those inoculated with vaccine alone (*p *< 0.05). No statistically significant differences were found between the control groups.

**Cytolytic T lymphocyte** **activity**

**Fig. 2:**
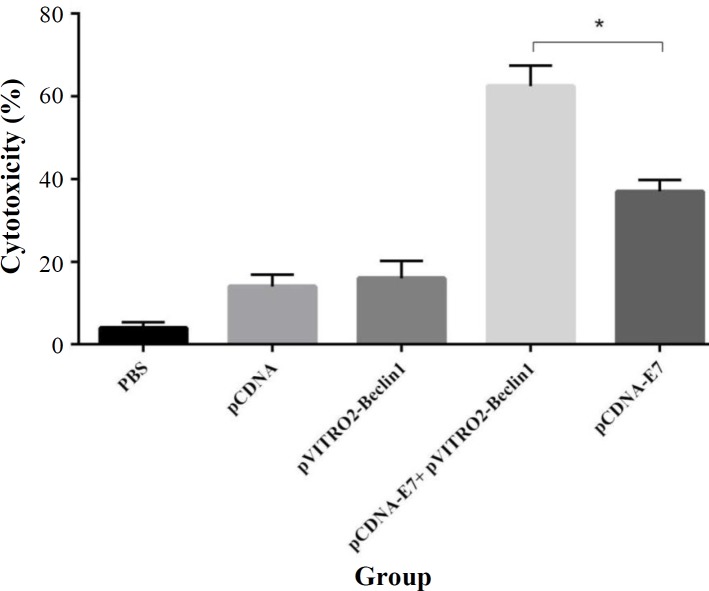
CTL activity. Each group of mice was immunized three times according to different groups. Two weeks after the last immunization, mice were sacrificed, and splenocytes were obtained. Then lymphocyte proliferation was performed using cytotoxicity detection kit. Results represented as the mean ±SD of five animals for each group. ^*^p < 0.05

In order to investigate the effectiveness of the vaccine to improve the E7-specific CD8 CTL response, the reaction in immunized mice was evaluated using the lactate dehydrogenase release assessment. As represented in Figure 2, HPV-16 E7 DNA vaccine enhanced the CTL response compared to the control groups (PBS, pCDNA,and pVITRO2-Beclin1). However, mice immunized with HPV-16 E7 DNA vaccine adjuvanted with Beclin-1 (pCDNA-E7 + pVITRO2-Beclin1) induced a higher cytotoxic response against E7 antigen than the E7 DNA vaccine group (*p* < 0.05).

**Cytokine assay**

**Fig. 3:**
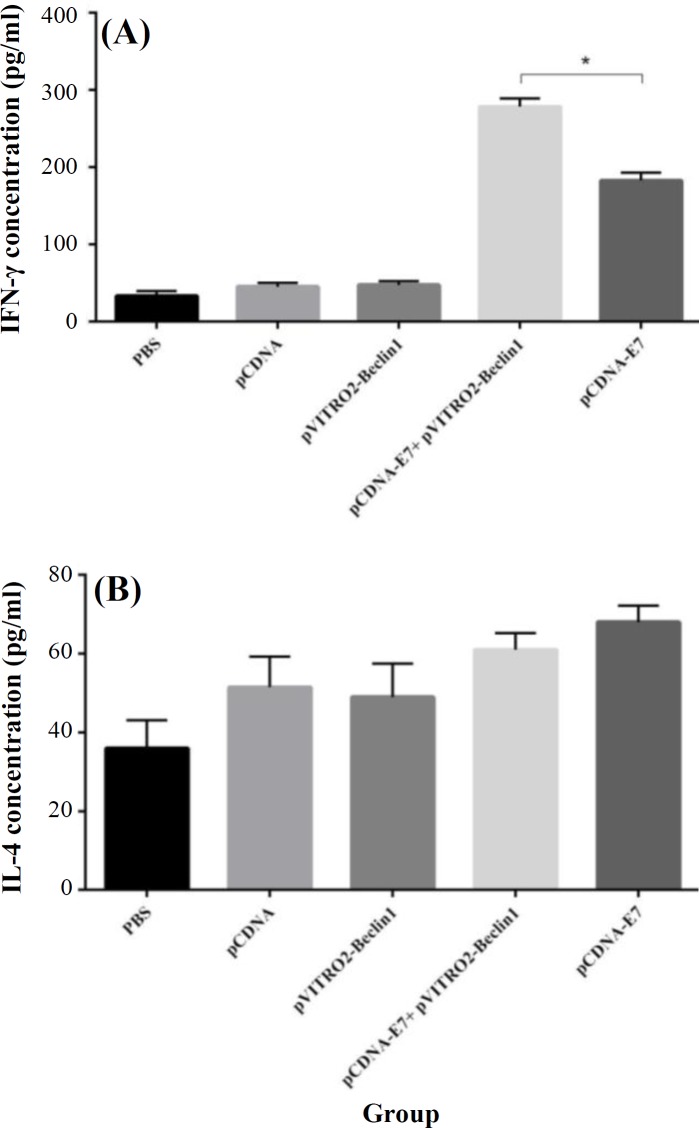
Cytokine assay. Each group of mice was immunized three times according to different groups. Two weeks after the last immunization, mice were sacrificed, and splenocytes were obtained. Then the expression levels of IFN-γ (A) and IL-4 (B) were performed using ELISA kit. Results represented as the mean ±SD of five animals for each group. ^*^p < 0.05

The splenocyte culture supernatants from the immunized mice were examined for E7-specific IFN-γ (as an indicator of Th1 response) and IL-4 (as an indicator of Th2 response) upon re-stimulation with antigen. As represented in Figure 3A, mice inoculated with HPV-16 E7 DNA vaccine adjuvanted with Beclin-1 (pCDNA-E7 + pVITRO2-Beclin1) produced significantly higher quantity of IFN-γ than mice vaccinated with DNA vaccine alone (*p* < 0.05). The new formulation non-significantly decreased the level of IL-4 as compared with HPV-16 E7 DNA vaccine (Fig. 3B). 

**Tumor regression**

**Fig. 4:**
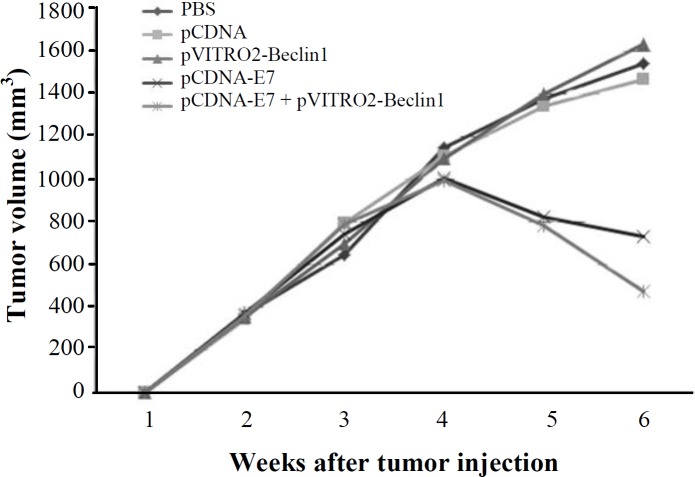
Tumor regression. The tumor size of immunized mice was evaluated up to six weeks. Tumor sizes represent the mean ± SD of 10 mice for four weeks and five mice after week four for each group. Line and scatter plot graphs depicting the tumor volume (in mm^3^) are presented

In order to assess tumor size by therapeutic inoculation, the mice that were challenged with 2 × 10^5^ TC-1 tumor cells were monitored twice a week following immunization for six weeks. As represented in Figure 4, in agreement with the increase in the E7-specific immunity by the novel adjuvant system, HPV-16 E7 DNA vaccine adjuvanted with Beclin-1 significantly reduced the tumor size when compared with the control groups. However, the tumor size was not remarkable in comparison with the pCDNA-E7 group.

## DISCUSSION

During the last years, several attempts have been made to promote the efficiency of immunostimulatory adjuvant system in vaccine development. Given the ability of autophagy to elicit humoral and cellular immunity, we hypothesized that co-formulation and co-administration of Beclin-1 (as an autophagy inducer) with HPV DNA vaccine can enhance antitumor effects. This process could promote DNA vaccine antitumor protective immune responses by inducing CD4^+^ and CD8^+^ T cells. To verify the hypothesis, we designed an *in vivo* experiment using a mouse tumor model. Mice were challenged with TC-1 tumor cells and immunized with a new formulation. Immunity was monitored as lymphocyte proliferation, CTL activity, cytokine assay, and tumor regression.

Autophagy-induced immunostimulators have been the subject of several studies, as they have shown reasonable ability to enhance MHC-I and -II antigen presentation^[^^25^^-^^30^^]^. Schmid *et al.*^[^^28^^]^ have shown that targeting influenza matrix protein 1 to autophagosome by fusion to LC3 (an autophagy marker) increases matrix protein 1 epitope presentation by MHC-II to CD4^+^ T cells. Another study has demonstrated that the fusion of the human immunodeficiency virus-1 antigen Gagp24 to p62 (an autophagy receptor) leads to the efficient antigen delivery into the autophagy pathway and increases the number of Gagp24-specific IFN-γ- producing T cells, suggesting a promising approach for vaccine development by autophagy induction^[^^29^^]^. Meerak *et al.*^[^^30^^]^ have reported that after subcutaneous immunization by a plasmid encoding mTOR-KD, as an autophagy-mediated vaccine in BALB/c mice, the highest levels of secreted IFN-γ and IL-2 were elicited when compared with the vaccine without mTOR-KD. These results suggest that a DNA vaccine regimen with autophagy induction stimulates primarily a Th1 immune response.

The current results clearly indicate that administration of HPV-16 E7 DNA vaccine adjuvanted with Beclin-1 when compared with vaccine alone induces: (a) higher E7-specific lymphocyte proliferation, (b) a strong E7-specific cytotoxic response, (c) greater IFN-γ production, and (d) reduction in tumor size. These observations are in agreement with the potential ability of Beclin-1 to induce CD4^+^ and CD8^+^ T cells^[^^31^^,^^32^^]^. The adjuvant induced lymphocyte proliferation and CD8^+^ T cell activity and shifted the CD4^+^ T cell response toward the Th1 not Th2. The induction of Th1 cytokines, but not Th2 cytokines, by autophagy induction has been reported previously^[31,32]^. Khateri *et al.*^[^^13^^]^ have reported that mice immunized with hepatitis E virus vaccine formulated with Beclin-1 display a humoral and cellular response. This observation has also been confirmed by Naziri *et al.*^[^^14^^]^. It has been reported that autophagy causes additional processing of the antigen, probably due to the engulfment of the antigen by autophagosomes and delivery to lysosomes for MHC-restricted antigen presentation^[^^33^^]^.

Although the exact reason for the immuno-stimulatory activity of autophagy induction has not been clarified, one possibility may be autophagy-mediated exogenous antigen processing for presentation by MHC-I through cross-presentation. The connection between autophagy and the delivery of intracellular and extracellular antigens to vesicular MHC-I-loading compartments has been determined^[^^34^^]^. Despite promising results, autophagy induction, as a novel strategy in the development of vaccines against infections, is still in the experimental stages, and the details of this mechanism remain to be fully elucidated.

Several adjuvants have been utilized in some researches for the assessment of HPV-16 E7, as a model antigen, in the improvement of a therapeutic DNA vaccine candidate. Our last studies have confirmed that HPV DNA vaccines expressing HPV-16 E7 adjuvanted with chitosan nanoparticles could enhance antitumor effects such as antigen-specific cytotoxic CD8^+^ T cell responses, IFN-γ production, and inhibition of tumor progression^[^^22^^,^^23^^]^. Induction of preservative antitumor immune response is caused by CD4^+^ T cells releasing Th1-type cytokines and CD8^+^ cytotoxic T cells^[^^35^^]^, similar to what was revealed by the current formulation. IFN-γ is a cytokine that plays a crucial role in antitumor host immunity and triggers a powerful antitumor effect by inducing Th1 polarization, CTL stimulation, and tumoricidal activity of dendritic cell^[^^36^^]^. The role of autophagy in cancer is complicated and probably is dependent on tumor type and stage. We need to know more about the role of this biological process in tumor regression; it seems obvious that modulation of autophagy would be a great promise for future cancer therapeutic approaches.

Taken together, the results of the current study clearly indicate that high antitumor immunity induction with Beclin-1 is a powerful tool in vaccine development and is a promising subject for further investigation. Furthermore, the simplicity, versatility, and biocompatibility of Beclin-1 immunotherapy make it a suitable candidate for clinical translation. Although these findings shed light on the application of autophagy induction as an adjuvant, further comprehensive investigations are warranted in this regard. Based on the results of this study, the potential induction activity of this formulation for factors such as antibody titration and other cytokine/chemokine levels would be interesting. Importantly, we used Beclin-1 and E7 on discrete plasmids. This separation largely resulted in the capture of the plasmids by various antigen-presenting cells that may impede the adjuvant effect. Thus, application of an adjuvant that is genetically fused to antigen and the use of carriers such as liposomes to entrap both plasmids inside a compartment and deliver them simultaneously to the cells could be helpful.

## CONFLICT OF INTEREST.

None declared.
